# Early Detection of Pediatric Type 1 Diabetes: The Expanding Role of Screening

**DOI:** 10.3390/children13020235

**Published:** 2026-02-07

**Authors:** Marco Calderone, Sara Aramnejad, Elèna Giliberto, Bruno Bombaci, Mariarosaria La Rocca, Arianna Torre, Fortunato Lombardo, Giuseppina Salzano, Stefano Passanisi

**Affiliations:** Department of Human Pathology in Adult and Developmental Age “G. Barresi”, University of Messina, 98122 Messina, Italy; marco.calderone@studenti.unime.it (M.C.); sara.aramnejad@studenti.unime.it (S.A.); elena.giliberto@studenti.unime.it (E.G.); mariarosaria.larocca@studenti.unime.it (M.L.R.); arianna.torre@studenti.unime.it (A.T.); fortunato.lombardo@unime.it (F.L.); gsalzano@unime.it (G.S.)

**Keywords:** antibodies, autoimmunity, children, HLA, population, prevention

## Abstract

Type 1 diabetes (T1D) is a common chronic autoimmune disease in childhood, often presenting abruptly and frequently complicated by diabetic ketoacidosis at diagnosis. T1D develops through well-defined presymptomatic stages characterized by islet autoimmunity and progressive dysglycemia, offering a window for early identification. This narrative review summarizes current evidence on screening for T1D in children and adolescents, focusing on target populations, screening strategies, and methodological approaches for autoantibody detection. Data from major international programs involving familial, high-risk, and general population screening are discussed, highlighting their impact on reducing diabetic ketoacidosis at onset, improving metabolic outcomes, and facilitating structured follow-up and family education. Advances in assay technologies, including electrochemiluminescence, multiplex platforms, and novel ultrasensitive methods, have enhanced the feasibility and accuracy of large-scale screening. The review also examines the public health implications, cost-effectiveness, and ethical considerations of implementing population-based screening, particularly in light of emerging disease-modifying therapies such as teplizumab. Overall, available evidence supports screening as a meaningful strategy to shift T1D diagnosis from an acute emergency to a predictable clinical trajectory, with potential benefits extending from individual patient outcomes to healthcare system sustainability.

## 1. Introduction

Type 1 diabetes (T1D) is the most common form of diabetes in the pediatric age, accounting for more than 90% of cases in Western countries. Globally, T1D is among the most frequent chronic diseases of childhood, with approximately 108,300 new cases in children and adolescents younger than 15 years reported in 2021 [1]. The median age at diagnosis in children varies by sex and geographic region, with a peak incidence during late childhood and adolescence, particularly between 10 and 14 years of age [2]. The etiology of T1D is multifactorial. In genetically predisposed individuals, environmental factors act as triggers, leading to chronic immune-mediated destruction of pancreatic β-cells and resulting in partial or, more commonly, absolute insulin deficiency. The high-risk HLA haplotypes for T1D are DR3-DQ2 and DR4-DQ8. The natural history of the disease is currently conceptualized as a staged process reflecting progressive autoimmune and metabolic dysfunction. Four stages of disease are currently recognized. Stage 1 is defined by the presence of multiple islet autoantibodies (IA), confirmed in at least two independent samples using validated assays, in individuals who remain normoglycemic and asymptomatic. Progression to stage 2 occurs when persistent multiple IA are associated with dysglycemia, which may manifest as fasting hyperglycemia, impaired glucose tolerance documented by oral glucose tolerance testing (OGTT), glycated hemoglobin (HbA1c) values between 5.7% and 6.5% (39–48 mmol/mol), or an increase in HbA1c of at least 10%. Within stage 2, individuals may be further characterized as stage 2a, corresponding to mildly elevated glucose levels, or stage 2b, in which glucose values approach the diagnostic thresholds for overt diabetes. Although these subcategories have been previously described, their formal designation provides additional descriptive clarity for both clinical practice and research settings. Stage 3 corresponds to the onset of overt diabetes, defined by hyperglycemia meeting the glycemic and clinical diagnostic criteria of the American Diabetes Association (ADA) [3], and may occur in either asymptomatic or symptomatic individuals. This stage can be further subdivided into stage 3a, encompassing asymptomatic individuals who nonetheless meet diagnostic glycemic criteria, and stage 3b, representing the classic clinical presentation with symptomatic hyperglycemia, including polyuria, polydipsia, and unexplained weight loss, necessitating immediate initiation of insulin therapy. Finally, stage 4 refers to established, long-standing T1D.

Autoantibodies specific for T1D include insulin autoantibodies (IAA), autoantibodies against glutamic acid decarboxylase (GADA/anti-GAD65), autoantibodies against the tyrosine phosphatase IA-2 (IA-2A/anti-IA-2), and autoantibodies against zinc transporter 8 (ZnT8A/anti-ZnT8). The presence of circulating autoantibodies increases the risk of progression to diabetes, with risk rising according to the number of autoantibodies detected, their titers, and the degree of β-cell dysfunction. The seroconversion is relatively common in early life, while the risk of developing multiple autoantibodies declines with increasing age [4,5].

Diabetic ketoacidosis (DKA) frequently complicates the presentation of new-onset T1D in children, with reported rates of 15–70% in Europe and North America, particularly among very young children and underserved populations, and in the context of delayed diagnosis. In pediatric patients with established diabetes, DKA is relatively infrequent and predominantly related to insulin omission or interruption of insulin delivery, with infections accounting for a minority of cases [6].

Each DKA episode in children with T1D is an independent risk factor for recurrence, with prior-year episodes markedly increasing the risk of subsequent DKA [7].

Early detection of islet autoimmunity and dysglycemia substantially decrease the risk of DKA at onset and contributes to more favorable metabolic trajectories after diagnosis. In this context, screening enables children and families to engage in anticipatory guidance, structured follow-up and potential immunomodulatory therapies capable of delaying disease progression. This review aims to provide a comprehensive overview of screening of T1D in children and adolescents, focusing on the target population, the methods of antibody identification, the goals of this campaign, and the cost–benefit ratio, through an analysis of the major international screening programs. Key terms such as “diabetic ketoacidosis”, “screening”, “Type 1 Diabetes”, “complications”, “prevention”, “assay”, “pediatric age”, “pediatrics”, and “children” were included in the search. Reviews, systematic reviews, meta-analyses, observational studies, randomized controlled trials (RCTs), and evidence-based guidelines of the major scientific societies published between 2005 and 2025 were included to capture the latest evidence and clinical insights. Three authors (MC, SA, EG) worked independently on three different online databases. Only studies published in the English language were considered for inclusion in this review. Additional sources were identified through the references of selected articles. Data were synthesized thematically. No formal meta-analysis was performed, given the narrative nature of the review.

## 2. The Concept of Screening

According to the paper published by the World Health Organization in 1966 [8], the principles related to screening are as follows:

- The condition sought should be an important health problem.
- The natural history of the condition, including development from latent to declared disease, should be adequately understood.
- There should be a recognizable latent or early symptomatic stage.
- There should be a suitable test or examination.
- The test should be acceptable to the population.
- There should be an agreed policy on whom to treat as patients.
- There should be accepted treatment for patients with recognized diseases.
- Facilities for diagnosis and treatment should be available.
- The cost of case-finding (including diagnosis and treatment of diagnosed patients) should be economically balanced in relation to possible expenditure on medical care as a whole.
- Case-finding should be a continuing process and not a “once and for all” project.

However, the evolution of medicine and healthcare systems has highlighted that the screening landscape is far more complex than originally envisioned. More recent revisions of the above-mentioned principles emphasize crucial aspects, including program acceptability by the population, the overall balance between benefits and harms, quality and performance management, and the organizational capacity of healthcare systems. Therefore, screening criteria should be considered as a dynamic balance between benefits and harms [9]. Screening is currently defined as a public health intervention aimed at the early identification of a disease or at-risk condition in apparently healthy individuals, before clinical symptoms appear [8,10]. It is not the simple execution of a diagnostic test, but a structured process that includes selection of the target population, test administration, communication of results, further diagnostic investigations, and management of positive cases [11]. In general, a population can be screened if there is evidence that early identification of the condition yields a public health benefit [12]. The primary objectives of screening are reducing disease-related mortality or morbidity, improving quality of life, and, in some cases, preventing disease onset through early intervention [9,13].

### 2.1. Objectives of Screening for Type 1 Diabetes

**Figure 1:**
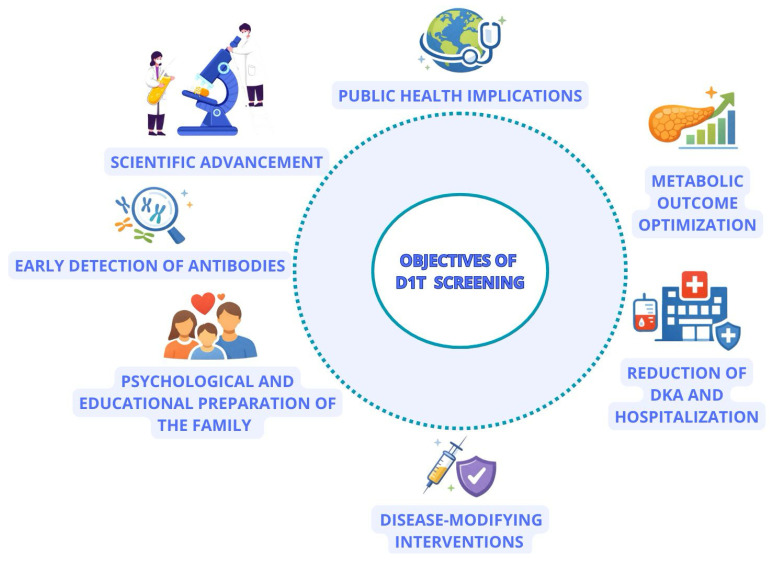
Objectives and benefits of pediatric type 1 diabetes (T1D) screening. Schematic overview of the main goals of islet autoantibody screening in children, including early detection of presymptomatic T1D, family education and psychological preparation, support for research, access to disease-modifying interventions, reduction in diabetic ketoacidosis and hospitalizations at diagnosis, optimization of metabolic outcomes, and broader public health benefits.

Screening for T1D in pediatric populations aims to shift the diagnosis from an acute metabolic emergency to a predictable and manageable event, thereby reducing morbidity and improving long-term metabolic outcomes. The main objectives of screening programs for T1D are: (1) to reduce the risk of DKA at onset by providing early diagnosis and monitoring; (2) to identify individuals eligible for preventive interventions or disease-modifying trials; (3) to organize monitoring and support pathways that minimize psychological impact and maximize follow-up safety (Figure 1) [14,15]. Large-scale experiences (such as population screening studies like Fr1da) have demonstrated the feasibility of detection using capillary/DBS (dried blood spots) sampling and the utility of the program in reducing adverse events at onset and recruiting participants for prevention studies [16]. A clinically and therapeutically pivotal development is represented by immunotherapy with Teplizumab, which has shown the possibility to delay progression to clinical T1D in stage 2 individuals; this makes screening even more relevant, as it creates tangible therapeutic opportunities for patients identified at early stages [17].

#### 2.1.1. Detection of T1D at the Earliest Disease Stage

The number of IA represents the strongest predictor of clinical progression. A single autoantibody does not define stage 1, but it is associated with a 15% 10-year risk of progression. When seroconversion occurs, it typically happens within a short interval, with a median time of 6.8 months [18]. The presence of two autoantibodies identifies preclinical T1D, conferring a markedly increased risk of progression: 74% of the patients develop stage 3 T1D within 10 years and 85% within 15 years, rendering eventual progression almost inevitable over the life course. Those at stage 2 similarly demonstrate a substantial risk, with approximately 75% advancing to stage 3 disease within 5 years [19,20]. The probability of progressions rises further to 92% in the presence of three autoantibodies. The specificity of autoantibodies further modulates progression risk: anti-IA2 carries the highest predictive value, followed by anti-ZnT8, IAA and anti-GAD [18]. The rate of progression to clinical T1D is also influenced by a combination of factors, including genetic background, autoantibody characteristics (such as number, type and titer), metabolic parameters (e.g., body mass index) and demographic variables (including age, sex and race/ethnicity) [21]. In this way, the primary aim of screening is to identify individuals with beta-cell-specific autoimmunity, allowing for a biological diagnosis of T1D prior to the development of clinical hyperglycemia.

However, despite its strong predictive value, autoantibody-based screening is not without limitations, and several sources of diagnostic uncertainty must be acknowledged. A proportion of children with clinically confirmed T1D present without detectable islet autoantibodies at diagnosis. Data from the “Diabetes Prospective Follow-up” (DPV) registry indicate that approximately 6–7% of pediatric patients are autoantibody-negative at onset, with higher rates observed in younger children [22]. Some of these children subsequently seroconvert, suggesting that early testing may yield false-negative results due to the temporal dynamics of autoimmunity. Technical variability further contributes to this uncertainty: comparative studies demonstrate discordance between assay platforms, reflecting differences in affinity detection and epitope recognition. Beyond analytical factors, interpretation of screening results poses an additional challenge. As shown in the ASK study, parental understanding of risk is highly heterogeneous, with frequent under- or overestimation of progression likelihood despite standardized counseling.

#### 2.1.2. Psychological and Educational Preparation of the Family

Screening provides families with a gradual educational pathway that mitigates the emotional impact of diagnosis and improves disease management competence. It also enables targeted intervention on modifiable risk factors, such as unhealthy diet and physical inactivity, which may accelerate progression to stage 3 T1D [18]. The Fr1da study showed that parents of children identified at stage 1 or stage 2 exhibited significantly lower depressive symptoms compared with parents of children diagnosed at stage 2 T1D who had not participated in prior screening [20]. Early and progressive comprehension of clinical information improves decision-making and prognosis. Parents frequently feel overwhelmed at diagnosis of T1D, so effective assimilation of relevant information enables families to initiate chronic disease management sooner, improving long-term outcomes in T1D. A key advantage of peer support lies in its ability to provide relevant, understandable information and practical strategies for managing the illness [23]. Preparation for insulin therapy, combined with structured education and psychological support, can alleviate caregivers’ anxiety and facilitate a smoother transition to stage 3 T1D and insulin treatment initiation.

At the same time, emerging evidence indicates that screening itself introduces a distinct psychological burden that must be acknowledged within these educational pathways. The ASK (Autoimmunity Screening for Kids) study demonstrated that notification of islet autoantibody positivity triggers clinically significant anxiety in the majority of parents, with more than 70% exceeding established thresholds for high state anxiety at the first follow-up visit. This heightened distress often persists over time and is disproportionately elevated among parents with lower educational attainment or from minority backgrounds, underscoring the influence of social determinants on psychological responses to screening. Moreover, parental risk perception is frequently inaccurate, with substantial variability in understanding the child’s true likelihood of progression. Integrating these findings with the broader benefits of early education suggests that screening programs must not only prepare families for future disease management but also actively mitigate the emotional consequences of early risk identification. Structured counseling, risk communication and ongoing psychosocial support should therefore be integrated as core components of screening and monitoring pathways to ensure that the psychological impact of early detection does not undermine its clinical advantages [24].

#### 2.1.3. Reduction in DKA and Hospitalization

Evidence from large research cohorts indicates that structured monitoring markedly reduces the incidence of DKA at onset and, consequently, the need for hospitalization. Several studies have shown that presentation with DKA in youth is associated with persistently higher HbA1c levels for up to 11 years after diagnosis. Absence of DKA at onset is also predictive of fewer severe hypoglycemic episodes a decade after diagnosis [14]. DKA is also increasingly recognized as having lasting consequences for metabolic control and neurocognitive function [25]. Screening for early-stage T1D significantly reduces the incidence of DKA at onset, decreasing morbidity, mortality and ultimately improving quality of life by reducing overall disease burden [26].

#### 2.1.4. Optimizing Metabolic Outcome After Diagnosis

Beyond preventing DKA, screening contributes to preservation of endogenous insulin secretion in the majority of children by enabling a more favorable clinical presentation at diagnosis [18]. The “Diabetes Control and Complications Trial” (DCCT trial) clearly demonstrated that achieving tight glycemic control through intensive therapy, since the very early weeks following the diagnosis, is associated with a substantially reduced risk of retinopathy progression, onset of microalbuminuria and severe hypoglycemic events [18,27,28]. Additionally, children identified with presymptomatic T1D through population-based screening show a markedly milder clinical profile at progression to stage 3 compared with unscreened people. Their presentation is characterized by lower HbA1c and fasting glucose levels, higher fasting c-peptide concentrations, fewer cases of ketonuria, a smaller proportion requiring insulin at diagnosis, a very low prevalence of DKA and BMI-SDS values within the normal range, likely reflecting minimal or absent weight loss [29]. Recent multinational evidence further reinforces the importance of avoiding metabolic decompensation at onset. In a cohort of over 9000 children from nine countries, those presenting with DKA, especially in its severe form, showed persistently higher HbA1c levels, greater insulin requirements, and higher BMI-SDS during the first two years after diagnosis, indicating a lasting metabolic disadvantage [30].

#### 2.1.5. Public Health Implications

One of the primary goals of the screening is to reduce the economic and clinical load of T1D through standardized protocols of early interception and targeted follow-up. Cost-effectiveness assessments must account for the short and long-term benefits of screening, including improved glycemic control and reduced morbidity. Moreover, with the emergence of interventions capable of delaying disease progression, the cost-effectiveness of screening must now be reconsidered from an additional future-oriented perspective [31,32]. The establishment of a national early-detection infrastructure represents a critical first step in enabling the evaluation of emerging therapies and in refining therapeutic algorithms [33]. Screening strategies should be adapted to various populations and global settings. Ethnic variation, genetic background and environmental factors can all influence the effectiveness of screening programs. Tailoring protocols to specific regions and populations maximizes accuracy and clinical relevance [34].

#### 2.1.6. Advancement of Scientific Knowledge

The opportunities for individuals to participate in research studies are an important aspect of screening programs. While screening for T1D provides clear benefits, potential risks and harms must also be carefully considered [20]. The objective is to generate data on the natural history of beta-cell dysfunction, on environmental factors and on the effectiveness of preventive interventions.

#### 2.1.7. Disease-Modifying Interventions

Early identification of children in the early stages of the disease allows access to immunomodulatory strategies that can modify the evolutionary trajectory of the T1D. Humoral immunity has been investigated as a potential preventive target in T1D, although clinical translation has remained limited. A phase II clinical trial evaluating rituximab, an anti-CD20 monoclonal antibody aimed at disrupting B-cell-mediated T-cell activation, demonstrated only modest and transient effects. Preclinical evidence suggested that neutralization of pro-inflammatory and Th1-associated cytokines, including tumor necrosis factor-α (TNF-α) and interleukin-1β (IL-1β), key mediators of immune-mediated β-cell injury, might modulate disease progression. However, subsequent clinical studies of cytokine-targeted therapies—such as etanercept (TNF-α inhibition), anakinra (IL-1 receptor blockade), and canakinumab (IL-1β neutralization)—failed to demonstrate consistent or clinically meaningful benefit. Similarly, abatacept (CTLA-4-Ig), which induces T-cell costimulatory blockade, showed immunomodulatory effects without clear evidence of sustained disease modification, underscoring the challenges of translating immunological rationale into durable clinical efficacy in T1DM prevention. Among immunomodulatory approaches, the anti-CD3 monoclonal antibody teplizumab has emerged as the most advanced therapeutic candidate to date, although its clinical benefit remains context-dependent and limited in scope [17].

Among the various immune and metabolism-based interventions studied, teplizumab is currently the only therapy approved by regulatory authorities to delay progression from stage 2 to stage 3 T1D in children aged >8 years [20,25,35]. Teplizumab is a humanized recombinant monoclonal antibody, which targets the CD3 chain on T lymphocytes, helping to preserve both β-cell and regulatory T-cell function, thereby maintaining endogenous insulin secretion [25]. Delaying the onset of T1D, teplizumab provides multiple benefits for patients, including reduced insulin requirements and hospitalizations, lower complication rates, and improved glycemic control [17]. Consequently, the progression to stage 3 T1D can be delayed by an average of approximately 2.7 years [36]. A recent phase 3 randomized, placebo-controlled trial demonstrated higher c-peptide levels in 217 children and adolescents treated with teplizumab, compared with placebo at 78 weeks after two 12-day treatment courses [36]. Adverse events are generally infrequent, early in onset and self-limiting, including mainly cytopenia (predominantly lymphopenia), aminotransferase elevations, skin rash and moderate flu-like syndrome attributable to cytokine release [18].

Despite its potential benefits, the clinical applicability of teplizumab remains restricted to a narrowly defined population, largely due to the absence of widespread screening programs for early-stage diabetes outside research settings. Moreover, the high cost of treatment represents a substantial barrier to broader implementation [20].

A survey-based analysis in 2023 found that only about half of parents and caregivers of youths at risk of developing T1D would consent to teplizumab treatment. Together with the limited awareness of this novel therapy, this finding highlights the need for targeted educational campaigns by healthcare societies, potentially supported by coordinated efforts between diabetes care providers and the media, as previously shown effective in DKA prevention [35].

## 3. Screening Strategies for Type 1 Diabetes

### 3.1. Screening of At-Risk Population

Currently, screening for T1D in pediatric age is restricted to individuals from categories at markedly higher risk of developing the disease. These include first-degree relatives of people with T1D, whose risk of developing the condition is 15–20 fold higher compared to the general population [37,38]. Concordance rates are reported to be 25–70% in monozygotic twins, 6–7% in dizygotic twins and siblings, and 1–9% in offspring of affected parents [39,40]. A retrospective study conducted by Urrutia et al. evaluated a cohort of 3015 first-degree relatives of patients with T1D, identifying antibody positivity in 48 individuals (1.59%) and observing progression to T1D in 54.2% of cases over a median of 5 years [37]. To uncover these latent cases, structured screening programs have emerged, specifically targeting first-degree relatives, such as the VISION-T1D project proposed in Saudi Arabia [41], the TrialNet Pathway to Prevention initiative [40] and the Diabetes Prevention Trial of Diabetes Type 1 (DPT-1) [42,43]. The preliminary results of the TrialNet Natural History Study demonstrated that the presence of multiple IA in relatives of individuals with T1D is a strong predictor of progression to clinical disease [44]. Autoimmunity precedes metabolic abnormalities by several years, confirming the existence of a prolonged preclinical phase and providing the rationale for screening programs and preventive interventions.

The DPT-1 was a secondary prevention study conducted in the United States and Canada from 1994 to 2003, aimed at evaluating whether parenteral or oral insulin could prevent or delay the onset of T1D in first- and second-degree relatives of affected individuals. Screening for the study was based on islet cell antibodies (ICA) testing and initially involved over 80,000 individuals. Those with ICA positivity (3483 subjects) underwent further staging, including assessment of HLA-DQ haplotypes, IAA status, first-phase insulin response during intravenous glucose tolerance testing, and glucose response to OGTT. Participants at high risk of developing diabetes (≥50% over 5 years) were eligible for the parenteral insulin trial, while those at intermediate risk (26–50% over 5 years) were eligible for the oral insulin trial. Randomization identified 339 and 372 participants for the respective trials, with 139 and 97 developing T1D, and no significant differences observed between the treatment and control groups [43,45].

Another subgroup at increased risk of developing T1D is represented by individuals with concomitant autoimmune diseases [39]. The conditions most frequently linked to T1D, and consequently requiring rigorous clinical and laboratory monitoring, include autoimmune thyroid diseases (AITD) and celiac disease (CD), followed by vitiligo, alopecia, dermatological disorders and rheumatologic diseases [46].

T1D and AITD are the most common autoimmune diseases worldwide, and numerous studies have highlighted the close link between these conditions [47,48]. For example, in the ASK study experience IA (GAD, IA-2, insulin, and ZnT8) were assessed in a large cohort of 31,968 children drawn from the general population in Colorado. Among the screened participants, 344 children had a previously diagnosed celiac disease and 2082 reported a family history of celiac disease. Among the 344 children with celiac disease, six were positive for multiple IA while seven had a single IA. In children with a family history of celiac disease (n = 2082), 25 were positive for multiple IA and 39 for a single IA. After adjustment for age and sex, the presence of multiple IA was significantly associated with celiac disease and with a family history of celiac disease compared to children without these characteristics [49].

### 3.2. Screening of the General Population

Approximately 90% of T1D diagnoses are made in individuals who do not belong to the aforementioned high-risk categories [33]. For this reason, in recent years the concept of extending T1D screening to the general population has gained increasing attention. Several screening programs across Europe and worldwide are currently evaluating the effectiveness and sustainability of population-wide screening approaches. These programs can be broadly divided into two categories: those based on birth cohorts, which involve genetic screening to identify neonates at increased lifetime risk of developing T1D, and those based on autoantibody detection, which are more costly if performed without prior genetic prescreening but provide greater specificity for stage 1 and stage 2 T1D [50].

#### 3.2.1. Birth Cohort Studies

HLA screening represents the basis of the TEDDY (The Environmental Determinants of Diabetes in the Young) Prospective cohort study, conducted from 2004 to 2025 in six clinical centers located in four countries (US, Sweden, Finland, and Germany), coordinated by the Health Informatics Institute, University of South Florida. Between 2004 and 2010, 425,104 newborns were screened and enrolled 8503 infants carrying high-risk HLA DR-DQ genotypes for T1D. Among these, 926 had a first-degree family history of the disease. Follow-up, initiated at a mean age of 3 months, consisted of quarterly visits during the first 4 years of life and biannual visits thereafter, with assessment of IAA, GADA, and IA-2A autoantibodies, and subsequently ZnT8, until the age of 15 years or earlier if a diagnosis of T1D was established. In children with one or more positive autoantibodies, an OGTT was performed every six months. By the end of October 2024, 917 children with persistent autoimmunity were identified, and 455 children were diagnosed with clinical T1D. The study also showed that the incidence of insular antibody positivity peaked at 9 months of age (32/1000/year) and decreased by the age of 5 years (10.1/1000/year) [51].

The DIPP study, a population-based prospective follow-up study conducted in three Finnish university hospitals since 1994, has screened more than 250,000 neonates by HLA typing using cord blood samples. In approximately 10% of cases, high-risk HLA genotypes were identified, and these children were invited to participate in follow-up programs until the age of 15 [50]. Islet autoantibodies (ICA as the primary screening tool for children born before 2003 and IAA, ICA, GADA and IA-2A for children born since 2003) were screened by serum samples at 3–12-month intervals until the age of 6 years and at 12–36-month intervals thereafter. By the end of December 2022, the DIPP study identified 1334 children with confirmed positivity for at least one IA and 560 children received T1D diagnosis during follow-up [52].

The BABYDIAB study is a longitudinal cohort conducted in Germany between 1989 and 2000 that investigated the natural history of T1D and pancreatic autoimmunity in 1650 children born to parents with T1D [53]. These children underwent venous blood sampling for the measurement of IAA, GADA, IA-2A, and ZnT8 autoantibodies during scheduled follow-up visits at 9 months and 2, 5, 8, 11, 14, and 17 years of age. In children who tested positive for any autoantibody, assessments were repeated every six months. During follow-up, a total of 152 children developed IA. Among them, 26 seroconverted at 9 months, 34 at 2 years, 38 at 5 years, 28 at 8 years, 14 at 11 years, 10 at 14 years, and 2 after 17 years of age. The study also confirmed a higher incidence of seroconversion at 9 months (18.5 [12.7–27] per 1000 person-years) and 2 years (21 [15.9–29] per 1000 person-years), followed by a decline at older ages (9.1 [6.3–12.5] per 1000 person-years at 5 years; 9.2 [5.7–11.7] at 8 years; 5.1 [3.1–8.5] at 11 years; and 6.9 [3.8–12.4] at 14 years). Furthermore, male children exhibited earlier seroconversion compared with females (9 months vs. 2 years). Finally, children carrying HLA DR3/4-DQ8 or DR4/4-DQ8 genotypes showed a significantly higher incidence of IA positivity between 9 months and 5 years of age.

GPPAD (Global Platform for the Prevention of Autoimmune Diabetes) is a European program carried out in centers across five countries (Germany, UK, Poland, Belgium, and Sweden), aiming to identify neonates or infants with a >10% risk of developing multiple T1D-associated autoantibodies by age of 6 years, in order to recruit them into a randomized controlled trial testing the efficacy of oral insulin versus placebo in preventing autoantibody seroconversion. Between October 2017 and December 2018, 50,669 infants were screened for high-risk HLA genotypes (DR3/DR4-DQ8 or DR4-DQ8/DR4-DQ8) [51]. By July 2021, more than 279,000 infants had been screened with a prevalence of increased genetic risk of 1.1% [50].

Risk stratification using a Genetic Risk Score has also been implemented in three recent U.S. screening initiatives: the Combined Antibody Screening for Celiac and Diabetes Evaluation (CASCADE) program, the Sanford Population Level Estimation of T1D Risk Genes in Children (PLEDGE) project, and the Precision Individualized Medicine for Diabetes (PRiMeD) project [50,53].

The Sanford PLEDGE study is a large-scale observational program initiated in 2020, based on population-level screening for T1D and CD autoantibodies, combined with genetic risk stratification at birth. The study aims to evaluate 33,000 children and adolescents aged 0–5 years, 9–16 years, and 6–17 years for first-degree relatives of individuals diagnosed with T1D. Screening procedures are fully integrated into routine pediatric visits conducted within Sanford Health facilities.

The PRiMeD study, conducted from May 2018 to March 2021, despite disruptions related to the SARS-CoV-2 pandemic, demonstrated the feasibility of population-based genetic screening for T1D. The program relied on genetic risk stratification using saliva samples collected from 3818 children and adolescents aged 2–16 years, recruited across 12 clinics in Virginia. The investigation identified 542 individuals classified as “high risk” (T1D GRS ≥ 5), of whom 28 underwent venous blood sampling for autoantibody testing (IAA, GADA, IA-2A, and ZnT8). Two children were subsequently diagnosed with stage 1 T1D [54].

#### 3.2.2. Autoantibody-Based Screening Programs

##### The FR1DA Study

The FR1DA study, conducted in Bavaria between February 2015 and March 2019, represents one of the largest population-based screening programs, having recruited 90,632 children aged 2 to 5 years. In this study, capillary blood samples were collected by primary care pediatricians and analyzed for the presence of GAD, IA-2A, and ZnT8 autoantibody using the 3-Screen Islet Cell Antibody ELISA (enzyme-linked immunosorbent assay). If the ELISA test yielded a positive result, the antibodies were retested, including IAA, through radio binding assays (RBA). In case of positivity for two or more autoantibodies, confirmation was required via venous blood sampling [31]. Through this approach, 261 children were identified as antibody-positive, corresponding to a diagnosis of presymptomatic T1D in approximately 0.3% of cases [33,55]. The study also assessed the economic impact of extending screening to the general population. Using a micro-costing approach, the estimated expenditure was €28.17 (95% CI 19.96–39.63) per child screened and €9177 (6460–12,827) per presymptomatic T1D diagnosis. Considering variability in costs related to disease prevalence and methodological approaches, and assuming a prevalence of presymptomatic T1D in Germany of 0.31%, the estimated cost would decrease to €21.73 (16.76–28.19) per child screened and €7035 (5426–9124) pre diagnosis [32]. To identify the onset of IA in the pediatric population beyond 5 years of age, starting from April 2019 the study, designated as Fr1da Plus, expanded recruitment to include children aged 1.75–10.99 years. To date, a total of 211,464 children has undergone screening, being performed at approximately 3 and 7 years of age. Among all screened children, 485 participated in metabolic staging. Of these, 360 were diagnosed with stage 1, 85 with stage 2, and 17 with stage 3 T1D. Among children classified as stage 1, 105 progressed to stage 3 over a median of 4.0 years (Interquartile Range 2.2–5.5). Among the 85 children identified at stage 2, 51 progressed to stage 3 [56].

Subsequent analyses from the Fr1da program have provided compelling evidence that population-based screening not only identifies children in the presymptomatic stages of type 1 diabetes but also influences the clinical phenotype at disease onset. In a comparative evaluation including 128 children who progressed to stage 3 after a prior early-stage diagnosis and 736 unscreened children from the DiMelli registry, those identified through screening presented with markedly attenuated metabolic derangement at diagnosis. Screen-detected children exhibited substantially lower HbA1c and fasting glucose levels, higher fasting C-peptide concentrations, and a reduced prevalence of ketonuria and DKA, alongside a lower need for insulin initiation. These differences were observed despite comparable age, sex, and genetic risk profiles, and remained evident irrespective of family history or the timing of diagnosis during the COVID-19 pandemic. Collectively, these findings indicate that early identification, coupled with structured education and monitoring, can meaningfully mitigate disease severity at clinical presentation [29].

##### The ASK Program

The Autoimmunity Screening for Kids (ASK) program, initiated in Colorado in 2017, aims to evaluate the costs and cost-effectiveness of extended screening in children and adolescents residing in the Denver metropolitan area [32]. To date, the study has screened more than 40,000 individuals aged 1–17 years [57] across private pediatric practices, private clinics, Children’s Hospital Colorado, and affiliated satellite sites. Screening targeted GAD, IAA, IA-2A, and ZnT8 autoantibodies using high-affinity radio binding assays (RBA) and electrochemiluminescence (ECL) methods. Children who tested positive on the initial screening underwent confirmatory retesting within 3 months. Those with persistent positivity for ≥1 autoantibody were invited to continue follow-up every 3–6 months, including clinical evaluations and HbA1c measurements. Children presenting multiple autoantibody positivity, concordant results across both assay platforms, or an HbA1c ≥ 6% underwent an OGTT and were offered optional biannual continuous glucose monitoring (CGM) [58]. In 2020, an analysis was conducted based on data from 10,029 children and adolescent collected up to July 2018, comparing two scenarios: the ASK program (including recruitment costs, questionnaires, research activities and negotiated laboratory fees) versus a routine scenario (excluding these components and applying the standard Centers for Medicare & Medicaid Services laboratory fee schedule). To assess the economic impact of the screening program, two models were employed.

The Bridge Model, designed to simulate the progression from diabetes risk (positivity for one or more autoantibodies) to the diagnosis of T1D. In this model, screening and follow-up costs are considered for all individuals screened up to age 18, with follow-up continuing until the age of 30.

The Lifetime Simulation Model, applied to individuals aged ≥30 years with a diagnosis of T1D, evaluates the impact of HbA1c modifications resulting from early diagnosis on both clinical and economic outcomes.

The estimated cost per screened individual was approximately 22.50 USD (United States Dollar). The overall cost of screening was 47 USD per child in the ASK program compared with 141 USD in the routine scenario. The cost per identified case was 4700 USD in ASK and 14,000 USD in routine screening. Using the Bridge Model, the total costs amounted to 560,000 USD and 1,641,000 USD for ASK and routine screening, respectively. According to the Lifetime Simulation Model, the estimated total costs were 19.5 million USD and 20.3 million USD. The authors concluded that, in order to achieve a cost-effectiveness threshold of USD/QALY (quality-adjusted life years) between 50,000 and 100,000 USD, the screening program would need to reduce the incidence of DKA at T1D onset by at least 20%, in conjunction with a sustained reduction in HbA1c of ≥0.1%. Screening would become cost-saving if DKA events were reduced by ≥40% and HbA1c decreased by ≥0.3% [32].

##### Protocol for the Australian Type 1 Diabetes National Screening Pilot

The pilot study conducted in Australia between July 2022 and June 2024 aimed to comparatively evaluate three potential models of population-based screening programs. The study established a maximum enrollment of 9000 participants, equally distributed across three cohorts:

- Cohort 1: Genetic risk-stratified neonatal screening using dried blood spot samples, with follow-up initiated at 11 months of age in those identified as being at risk of disease.
- Cohort 2: Genetic risk-stratified screening using saliva samples collected at 6–12 months of age, with follow-up beginning at 10 months of age for those at risk.
- Cohort 3: Autoantibody-based screening using capillary blood collected on filter paper at ages 2, 6, and 10 years, or at 5–6 years and 9–10 years when performed in the school setting.

The rationale for comparing these different screening modalities lies in the advantages of autoantibody-based screening for confirming disease status. Moreover, the majority of the population will test negative. However, to achieve a sensitivity approaching 80%, given that autoantibodies may appear at any age, testing must be repeated at least twice during childhood. Conversely, genetic risk-stratified assessment provides lifelong risk information and, when performed at birth, enables the identification of individuals at risk even before the age of two years [50,59].

##### Study Protocol D1Ce Screen

According to the approval of Law 130/2023, Italy became the first country worldwide to enact legislation promoting population-based screening for T1D and CD [21,60], with the aim of reducing the burden of these conditions. The project places the primary care pediatrician at the center of the screening process, assigning them an active role in case identification and referral to regional reference centers in the event of a positive screening result [61]. The initiative was preceded by the D1Ce pilot study, coordinated by the Italian National Institute of Health (ISS), which aimed to evaluate the feasibility, sustainability and acceptability of the screening program as outlined in the legislation. This multicenter observational study, conducted in four regions (Lombardy, Campania, Marche and Sardinia), screened 5363 children (representing 1.6% of the Italian pediatric population) for T1D-specific autoantibodies across three age groups: 2–2.9, 6–6.9, and 10–10.9 years, during the period between May 2024 and March 2025. Primary care pediatricians played a pivotal role in the project, being responsible for patient enrollment, informing families about the study, performing capillary blood sampling and sending specimens to the designated laboratory. Individual kits were distributed to family pediatricians, each containing single-use lancets, 200 µL microtubes for capillary blood collection and Guthrie/dried blood spot cards. All samples were centralized at a single laboratory (Laboratory of Autoimmunity, Diabetes Research Institute and Laboratory of Clinical Genomics, IRCCS San Raffaele Hospital, Milan), where they were analyzed for T1D-specific autoantibodies [60].

In 2025, the Diabetes Study Group of the Italian Society of Pediatric Endocrinology and Diabetology (ISPED) conducted a nationwide survey among pediatric diabetes centers to assess the frequency of DKA at T1D diagnosis between 2023 and 2024. Over two years of observation, T1D was diagnosed in 2398 children and adolescents. Of these, 821 (34.2%, 95% CI: 32.3–36.2) presented with DKA at onset, and 316 (13.2%, 95% CI: 11.9–14.6) with severe DKA [62].

Furthermore, when comparing regions included in the D1Ce pilot project with those not participating, a higher incidence of DKA was documented in the latter group. In pilot regions, the risk of DKA was reduced by 26%, and the risk of severe DKA was reduced by 49%. This reduction was observed both during the implementation of the screening program and prior to its initiation, being known as the “awareness effect”. Although it is not easy to accurately assess the costs and benefits of a population screening program before it is actually implemented, considering the approximately 1800 new annual diagnoses of T1D in Italy and the incidence of DKA at onset of 37% (data observed in the control regions), the awareness effect was able to prevent about 170 cases of DKA before the implementation of the D1Ce protocol, generating a gross cost saving of about €400,000 against an expenditure (including webinars, educational meetings, and central administrative overhead) of about €250,000–€350,000 [63]. However, the true economic burden of large-scale population screening remains uncertain until such programs are implemented in routine clinical practice. A comprehensive assessment of costs, including the diversion of pediatric primary care resources, strain on laboratory infrastructure, and the potential burden of psychological follow-up, will be essential to accurately determine the cost–benefit balance.

## 4. Methods for the Detection of Islet Antibodies

**Figure 2:**
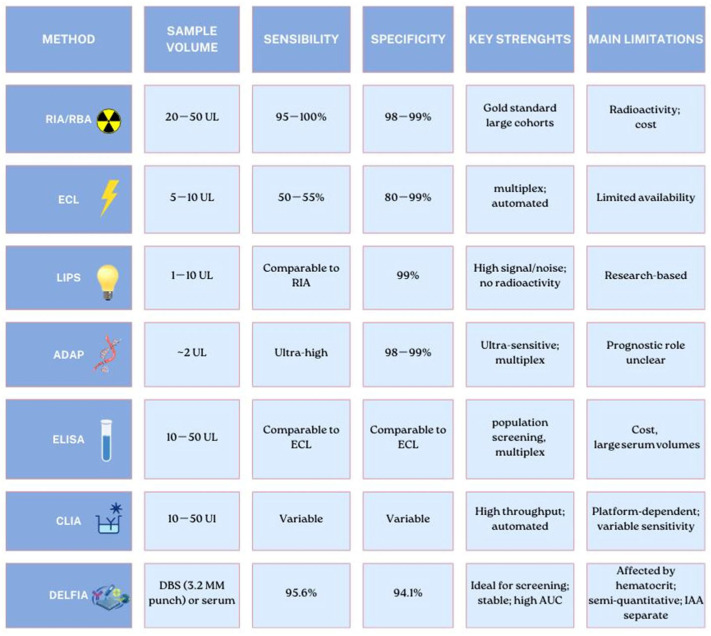
A summary of the main differences among methods for the detection of islet autoantibodies. For each technique, sensitivity and specificity values are reported, along with strengths and limitations.

In recent decades, scientific advances combined with the wider implementation of screening programs have improved the detection of autoantibodies, reduced the blood volume required for testing and increased the specificity [64]. To date, radioimmunoassay (RIA) has been considered the gold standard for autoantibody detection because of its good sensitivity and specificity. However, RIA detects both disease-relevant and non-disease-associated signals, and its technical complexity, the limited shelf-life of radioactive tracers, and high costs have driven the development of numerous innovative methodologies that offer valid alternatives to traditional assays and create new opportunities for early diagnosis and clinical investigation (Figure 2) [64,65]. Developments in the field have not only improved sensitivity and specificity but have also replaced radioactive tracers with non-radioactive detection systems [64].

### 4.1. Electrochemiluminescence (ECL)

In electrochemiluminescence-based assays, the antigen immobilized on the plate surface interacts with autoantibodies present in the sample, which in turn bind a second antigen conjugated to a luminescent label. Signal emission occurs when bivalent IgG bridges the two antigens [64,65].

In a study by Miao et al. [66], IAA and GADA autoantibodies were measured by ECL assay in a cohort of 3484 relatives enrolled in the TrialNet Pathway to Prevention study (PPS). Sensitivity, specificity, and predictive value were compared with those obtained by RAD-IAA and RAD-GADA. The results showed that the sensitivity of ECL-IAA (54.7%) was comparable to that of RAD-IAA (48.4%), and specificity was similar for ECL-GADA (79.2%) and RAD-GADA (76.8%). Both ECL-IAA and ECL-GADA were able to discriminate against individuals at high risk of progression to T1D. Moreover, most subjects who were single-positive by RAD-IAA or RAD-GADA tested negative by the corresponding ECL assays. Of RAD-IAA-positive subjects who were ECL-IAA-negative, 51.1% lost antibody positivity during follow-up (median 1.5 years); similarly, 62.9% of individuals with single RAD-GADA positivity but negative ECL-GADA lost their positivity. These findings indicate that ECL-IAA and ECL-GADA detect fewer disease-irrelevant signals than RIA [67], and that such patients, who have a lower risk of progressing to overt diabetes, may be candidates for less intensive monitoring [68]. The method also allows simultaneous measurement of multiple autoantibodies when specific multiplex assays are employed. In the study by Zhao et al. [69], sera from 40 newly diagnosed T1D patients recruited at the Barbara Davis Center for Childhood Diabetes and 50 healthy controls were randomly selected. All T1D patients were positive for at least one autoantibody, and 19/40 were TGA (Tissue Transglutaminase IgA antibodies)-positive by RIA. Using the QuickPlex4-Spot plate, four distinct autoantibodies (mIAA, GADA, IA-2A and TGA) were assayed with 6 µL of blood per well; results were compared with standard RIA and validated ECL assays. The study found that the multiplex method achieved 100% specificity in healthy controls and demonstrated superior detection of IAA and TGA compared with RIA, with potential signal interference observed in only 3 out of 180 measurements.

### 4.2. Luciferasi Immuno Precipitation System (LIPS)

The LIPS assay employs antigenic proteins fused to luciferases, enzymes originally derived from marine organisms, to detect the presence of autoantibodies. The antigen–luciferase fusion, mixed with a defined small volume of serum (1–10 µL), forms immune complexes that are captured on immunoglobulin-coated beads (Protein A/G binding the Fc region of IgG). Multiple wash steps remove unbound antigen from the supernatant, after which luminescence is measured and expressed in lights units (LU). The measured signal is directly proportional to the amount of antibody present in the sample. This method has several features that make it well suited for autoantibody detection and profiling, including rapid synthesis of recombinant antigen-luciferase fusion proteins, a high signal-to-noise ratio, a wide dynamic range for antibody detection, and the absence of radioactive labels in the fusion proteins [70].

In T1D, the LIPS method represents a valid alternative to the radioimmunoprecipitation assay (RIP). Comparative studies have shown that LIPS and RIP exhibit similar sensitivity and specificity for the principal T1D-associated autoantibodies [71]. Moreover, NanoLuciferase (Nluc)-IAA LIPS assays have demonstrated not only high concordance with RBA but also enhanced sensitivity for detecting new-onset T1D and predictive utility in identifying first-degree relatives who subsequently progressed to T1D [72]. In a study by Wyatt et al. [73], sera from 154 recent-onset T1D patients and 732 first-degree relatives were evaluated; among the relatives, 271 were RBA-GADA positive and 64 subsequently developed diabetes. All samples were assayed by the harmonized RBA protocol using full-length 35S-GAD65(1–585) and N-terminally truncated 35S-GAD65(96–585) antigens, and results were compared with a LIPS platform employing NanoLuc-tagged GAD65 constructs (Nluc-GAD65(1–585) and Nluc-GAD65(96–585)). The sensitivity of the Nluc-GAD65 assay was comparable to that of 35S-GAD65(96–585) in both patient and high-risk relatives. Moreover, LIPS using the Nluc constructs demonstrated higher specificity than the RBA based on 35S-GAD65(1–585) and/or 35S-GAD65(96–585). Similarly, detection of ZnT8 autoantibodies by LIPS has yielded favorable results. Using a fluid-phase LIPS immunoassay based on a NanoLuc-tagged dual heterodimer (ZnT8-R325 + ZnT8-W325; Nluc-ZnT8), investigators observed high concordance between LIPS and RBA, and comparable overall diabetes risk among first-degree relatives who tested positive or negative by both assays [74].

### 4.3. Multiplex Antibody Detection by Agglutination PCR (ADAP)

The antibody detection by agglutination-PCR (ADAP) is an ultrasensitive, solution-phase method for antibody detection [75]. In this assay, antibodies bind to and agglutinate synthetic antigen-DNA conjugates, bringing the DNA strands into proximity and enabling their ligation, followed by quantification through quantitative PCR. This approach allows detection of zepto- to attomole quantities of antibodies from only 2 μL of sample, with a dynamic range spanning 5–6 orders of magnitude. In addition, the multiplexing capability of ADAP enables the simultaneous measurement of multiple antibodies within a single assay [75].

Performing the assay entirely in solution preserves the antigen’s native conformation and eliminates the need for washing or centrifugation steps to remove unbound reagents, thereby markedly enhancing sensitivity while requiring only minimal deviations from a standard PCR workflow [75]. Given that T1D is characterized by the presence of multiple autoantibody biomarkers, a barcoded, multiplex approach is particularly advantageous for their concurrent detection in a single test [75]. ADAP demonstrates a sensitivity of approximately three orders of magnitude higher than that of clinically used assays, offering new opportunities for early disease detection and intervention [76]. As a no-wash assay, ADAP avoids the labor-intensive optimization of washing and centrifugation steps needed to preserve low-affinity antibodies in other platforms. It also prevents the requirement for unique monoclonal antibodies, as needed in some ELISA formats and does not rely on animal-derived antibodies as capture reagents, thereby eliminating interference from patient heterophilic antibodies [76].

ROC AUC analyses showing no significant differences between ADAP and RBA for GADA and IAA indicate that these assay formats are comparable for these autoantibody targets [76]. The early-appearing IA highlighted in longitudinal studies such as TEDDY, DIPP, and the T1DI consortium underscore the need for highly sensitive assays, particularly for IAA and GADA. The sample-sparing nature of multiplex ADAP is therefore especially valuable. Moreover, ADAP enables investigation of autoantibody subclasses; for example, preabsorbing sera with protein A–Sepharose, which does not bind IgG3, may allow specific identification of IgG3-GADA, IgG3-IA-2A, and IgG3-ZnT8A [76]. Overall, the 5-plex ADAP assay, designated for the simultaneous detection of IAA, GADA, IA-2A, ZnT8-A and TGA, shows sensitivity and specificity comparable to established methods at the time of clinical diagnosis of childhood T1D [76]. Importantly, ADAP demonstrates increased diagnostic sensitivity and specificity for IAA, GADA, and ZnT8A, although this improvement is not observed for IA-2A. In addition, ADAP is able to detect additional positive samples that may be missed by traditional assays [76]. Nevertheless, despite its technical strengths, ADAP has not yet demonstrated superiority over RIA or ECL in predicting which individuals are most likely to progress to clinical disease [64].

### 4.4. Enzyme-Linked Immunosorbent Assay (ELISA)

The EIA/ELISA platform is based on the fundamental immunological principle that an antigen binds to its specific antibody, enabling the detection of minute quantities of antigens, including proteins, peptides, hormones or antibodies, in fluid samples [77]. These assays employ enzyme-labeled antigens or antibodies to detect the target biomolecules, with alkaline phosphatase and glucose oxidase among the most commonly used enzymes. Typically, the antigen in liquid phase is immobilized in 96-well microtiter plates and allowed to bind to its specific antibody. This interaction is then detected using an enzyme-secondary antibody. The addition of a chromogenic substrate triggers a reaction that produces a visible color change or fluorescence, indicating the presence of the antigen. Both quantitative and qualitative measurement can be derived from the resulting colorimetric signal, while fluorogenic substrates provide enhanced sensitivity and allow precise quantification of antigen concentration in the sample [77,78]. Although not a novel approach, ELISA has demonstrated sensitivity and specificity comparable to the ECL assay, as it relies on similar autoantibody-capture principles and therefore shares many of its advantages [64]. Historically, its main limitations were cost and requirement for relatively large serum volumes. These challenges have been largely addressed through multiplexing, enabling simultaneous detection of GADA, IA-2A and ZnT8A [64,79].

This strategy has been successfully implemented as a first-line screening tool in the Fr1da population-based study in Germany, with RIA used as a second-line confirmatory test for samples that tested positive in the multiplexed screen [64]. Remarkably, even when the titers of the individual autoantibodies are below the detection limit of the assay, a cut-off index value of ≥14 in the 3 Screen ICA ELISA may still identify very low levels of emerging autoimmunity against pancreatic β cells, facilitating the early recognition of individuals at increased risk for T1D [79].

### 4.5. Dissociation-Enhanced Lanthanide Fluorescent Immunoassay (DELFIA)

DELFIA-based assays represent an immunodetection method relying on time-resolved fluorescence (TRF) using lanthanide chelates (Europium, Terbium, Samarium) as labels. Owing to their long decay times (in the millisecond range), these chelates allow measurement of the emitted signal after a delay following excitation, when matrix autofluorescence has already dissipated, thereby reducing background noise. In a study conducted by Dufrusine et al. [80], serum samples and dried blood spots (DBS) were collected from 68 patients with T1D and 51 pediatric controls with the aim of developing and validating a DELFIA-based multiplex method for the measurement of three islet autoantibodies (GADA, ZnT8, and IA-2A). Serum samples were analyzed using a validated ELISA method, whereas DBS were analyzed using the multiplex DELFIA assay. The results demonstrated a high concordance between the two methods (Spearman correlation r = 0.96, *p* < 0.0001) and confirmed excellent stability of DBS samples over time, supporting the potential use of this approach in population-based screening programs.

### 4.6. Chemiluminescent Immunoassay (CLIA)

CLIA is an immunoassay that employs a luminescent molecule as a label for the analytical reaction, capable of producing a light signal, upon chemical activation, proportional to the amount of antibody present in the sample. In automated systems, the serum sample is incubated with a labeled antigen and magnetic microparticles. Following the formation of the labeled antigen–antibody complex, the system separates the bound fraction from the free fraction. Finally, the reagents required to trigger the chemiluminescent reaction are added. In a study conducted by Danese et al. [81], 104 serum samples were collected from children and adolescents with recent-onset T1D and from first-degree relatives of individuals with T1D. Each serum sample was divided into two aliquots, used respectively for ELISA and CLIA testing. GADA, IA-2A, and ZnT8 autoantibodies were measured using both methods. The study showed a strong correlation between the two methods for GADA and a moderate correlation for IA-2A and ZnT8. However, compared with ELISA, the CLIA method tended to underestimate GADA and ZnT8 antibody concentrations and to overestimate IA-2A.

## 5. Conclusions

Screening strategies enable the identification of presymptomatic stages of T1D, thereby significantly reducing the incidence and severity of DKA at disease onset and promoting a more favorable metabolic trajectory following diagnosis. Beyond clinical benefits, screening provides families with a valuable opportunity for psychological preparation and appropriate educational training, improving acceptance of diagnosis and long-term disease management. The availability of disease-modifying therapies, such as teplizumab, has further redefined the role of early diagnosis, transforming screening from a purely preventive tool into a true gateway to therapeutic intervention.

Available evidence supports an overall favorable cost–benefit ratio of screening programs, particularly when considering reductions in acute complications and improvements in long-term glycemic control, despite their substantial economic impact. In this context, the implementation of population-based screening initiatives represents a landmark step toward integrating early diagnosis of T1D into healthcare systems, while simultaneously providing a real-world framework for evaluating clinical effectiveness, organizational feasibility, and economic sustainability. Future objectives include optimizing test performance, refining risk stratification models, and adapting screening protocols to different populations.

## Data Availability

No new data were created or analyzed in this study.
